# Paratesticular myofibroblastic lesion in a prepubertal child: a case report of a rare spindle cell tumor with testis-sparing management

**DOI:** 10.3389/fruro.2026.1866045

**Published:** 2026-08-21

**Authors:** Jeremy Sheiber, Jordan Nguyen, Michelle Smith, Manish Bajaj, Abhishek Seth, Pamela Ellsworth, Peter Y. Cai

**Affiliations:** 1 University of Central Florida College of Medicine, Orlando, FL, United States; 2 Division of Urology, Department of Surgery, Nemours Children’s Hospital, Orlando, FL, United States

**Keywords:** case report, myofibroblastic tumor, paratesticular mass, pediatric urology, testis-sparing management

## Abstract

Paratesticular tumors are uncommon in the pediatric population and can represent a wide range of benign and malignant pathologies. We report the case of an otherwise healthy 2-year-old boy who presented with an asymptomatic right scrotal mass. Serial scrotal ultrasounds demonstrated a stable, well-circumscribed extratesticular lesion adjacent to the epididymal head with internal vascularity and normal bilateral testicular architecture. Serum tumor markers, including alpha-fetoprotein, beta-human chorionic gonadotropin, and lactate dehydrogenase, were within normal limits. There was no family history of malignancy or urologic conditions. Given persistent diagnostic uncertainty, surgical exploration was performed. Intraoperatively, a firm paratesticular mass arising from the tunica albuginea was identified and excised with preservation of the testis. Frozen section analysis revealed bland spindle cells without malignant features, allowing for testis-sparing management. Permanent histopathology demonstrated a well-circumscribed spindle cell lesion within a loose collagenous stroma with dystrophic calcifications. Immunohistochemistry showed positivity for smooth muscle actin and negativity for desmin, myogenin, MyoD1, β-catenin, S100, and anaplastic lymphoma kinase (ALK), supporting a diagnosis of benign myofibroblastic lesion, such as fibrous pseudotumor, and excluding inflammatory myofibroblastic tumor. To our knowledge, this case represents one of the earliest presentations of a benign paratesticular myofibroblastic lesion. This case underscores the importance of considering rare benign spindle cell lesions in the differential of paratesticular lesions in young children and the use of intraoperative frozen section to facilitate testis-sparing surgery and avoid unnecessary orchiectomy through careful multidisciplinary evaluation.

## Introduction

Paratesticular tumors are uncommon in the pediatric population, with prepubertal testicular and paratesticular neoplasms accounting for approximately 1%–2% of all solid tumors in children and an estimated incidence of 0.5–2 cases per 100,000 (1). Prepubertal testicular and paratesticular tumors demonstrate a peak incidence at approximately 2 years of age, with up to 60% presenting before this age (1). Unlike intratesticular masses, which are more frequently malignant, the majority of paratesticular lesions in children are benign (1, 2). Although registry studies suggest that approximately 40% of childhood testicular tumors are benign, detailed characterization of paratesticular myofibroblastic and fibrous lesions is lacking (1).

Among benign paratesticular lesions, fibrous pseudotumor is reported as the third most common entity after adenomatoid tumor and lipoma (2). While fibrous pseudotumor has been well described in adults, pediatric cases are exceptionally rare, with only a handful reported beyond isolated case reports (6). Similarly, inflammatory myofibroblastic tumors (IMTs) of the paratesticular region have been described predominantly in adolescents, further limiting comparative pediatric data (6, 7). As a result, the differential diagnosis of paratesticular spindle cell lesions in very young children remains challenging.

Myofibromas represent the most common fibrous tumor of childhood overall with majority presenting during infancy and childhood, yet testicular myofibromas are extremely rare with only three previously reported cases in the pediatric population (3–5). Involvement of paratesticular soft tissues specifically is exceptionally rare and has not been reported in the literature (3). This rarity and lack of characteristic ultrasound and pathologic findings additively contribute to diagnostic uncertainty, particularly when distinguishing neoplastic from reactive fibrous lesions. Myofibroblastic lesions may mimic malignant tumors clinically and radiographically, historically leading to unnecessary radical orchiectomy (5, 6). Recognition of these rare entities and their distinguishing histopathologic features is therefore critical to facilitate testis-sparing management.

Here, we report a case of a paratesticular spindle cell lesion in a 2-year-old child, highlighting the diagnostic considerations and histopathologic distinction between benign myofibroblastic lesion and IMT, and review the relevant literature to emphasize implications for surgical decision-making.

## Patient background

The patient is a 2-year-old fully vaccinated boy with no significant past medical history, social history, surgical history, or family history who initially presented to the emergency department accompanied by his mother with concern for right scrotal swelling. The mother reported palpating a small, firm lump near the right testicle while changing the patient’s diaper one day prior to presentation. The lesion appeared less prominent the following day but remained palpable. The patient was asymptomatic and without associated fever, pain, hematuria, overlying skin changes, urethral discharge, gastrointestinal symptoms, or constitutional complaints. There was no known family history of genitourinary malignancy.

Initial scrotal ultrasound with Doppler demonstrated a 1.2-cm isoechoic extratesticular lesion in close proximity to the right epididymal head with internal vascularity. The right and left testes were normal in size and echotexture without intratesticular masses. Arterial and venous blood flow to both testes was normal. A trace right hydrocele was noted. The differential diagnosis at that time included torsed appendix testis and less likely a supernumerary testicle, and follow-up imaging was recommended.

The patient was subsequently referred to pediatric urology for further evaluation. Serum tumor markers including alpha-fetoprotein (AFP), beta-human chorionic gonadotropin (β-hCG), and lactate dehydrogenase (LDH) were obtained and were within normal limits.

**Figure 1:**
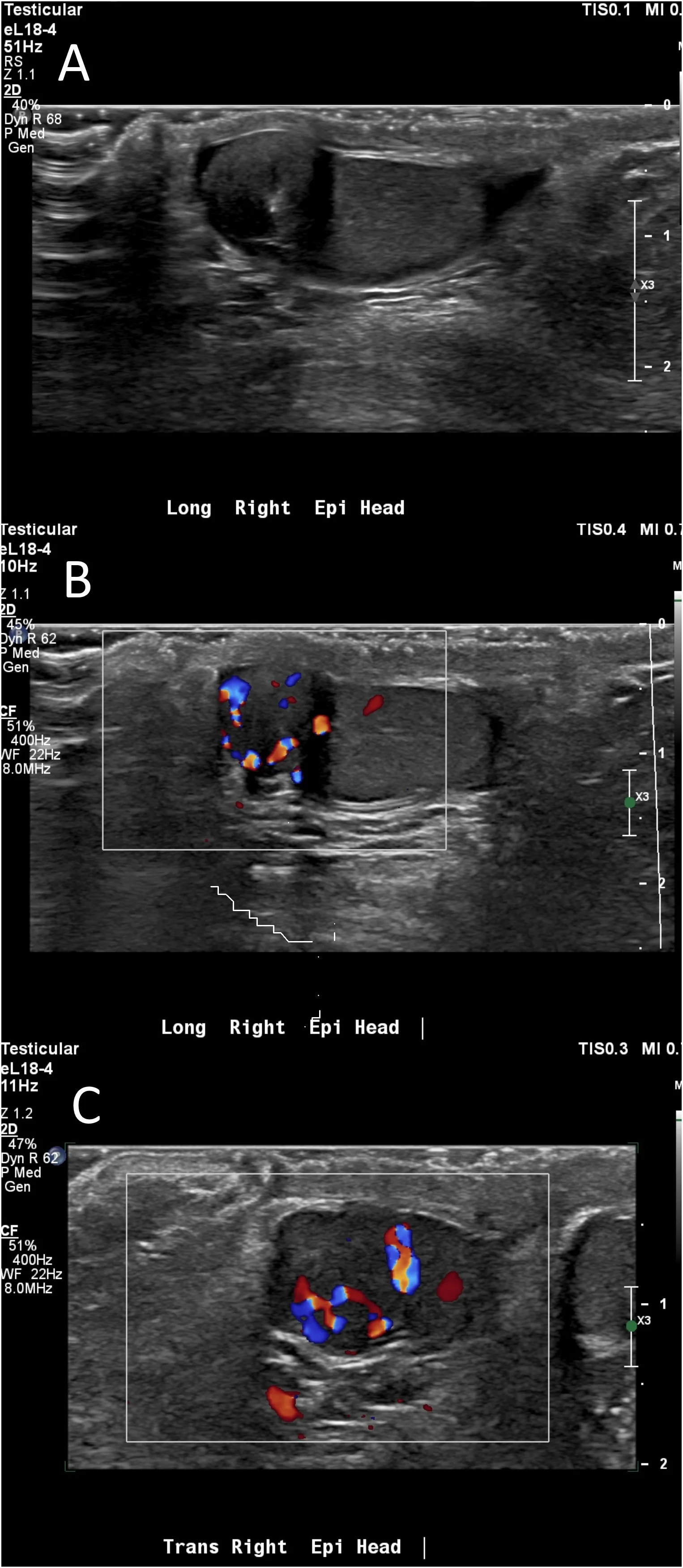
Round hypoechoic extratesticular mass along the right epididymis **(A)** with increased blood flow measuring 0.9 × 1.0 × 1.0 cm **(B, C)**.

Over the ensuing 3-month period, the patient underwent serial scrotal ultrasounds with Doppler. These studies consistently demonstrated a stable, well-circumscribed extratesticular mass measuring approximately 0.9–1.1 cm adjacent to the superior aspect of the right testicle, with internal vascularity and echogenicity similar to testicular tissue (**Figure 1**). At times, the lesion demonstrated a central echogenic focus suggestive of a mediastinum-like structure, raising consideration for a supernumerary or bilobed testicle. No interval growth or concerning radiographic changes were observed. Bilateral testes remained normal in size and echotexture with preserved symmetric blood flow. A small right hydrocele persisted, with trace left hydrocele noted on later imaging. No inguinal canal or spermatic cord abnormalities were identified.

On preoperative evaluation, the patient remained asymptomatic and was otherwise healthy with no new diagnoses or complaints. Physical examination revealed a normal circumcised phallus with bilaterally descended testes. A firm, smooth, approximately 1-cm mobile mass was palpated superior to the right testicle. Review of serial imaging confirmed a stable right extratesticular lesion.

Given the lesion’s stability and negative tumor markers, the mass was felt to be most likely benign; however, malignancy, particularly paratesticular rhabdomyosarcoma, could not be definitively excluded. Management options including continued surveillance versus surgical exploration and excision were discussed in a multidisciplinary setting and subsequently with the patient’s family. Given uncertainty about pathology and parental concern, a decision was made to proceed with surgical exploration with intraoperative frozen section.

## Operative course/pathology analysis

After informed consent, the patient was taken to the operating room and placed supine under general anesthesia. A right inguinal incision was made, the spermatic cord was isolated, and proximal control was established as is typically done for malignancy. The right testis was delivered, revealing a firm extratesticular mass at the superior pole within the tunica vaginalis, in close proximity to the spermatic cord. The lesion was carefully dissected free from surrounding structures and noted to be attached by a thick stalk to the tunica albuginea. The stalk was excised with a 2- to 3-mm margin of tunica albuginea, and hemostasis was achieved with bipolar cautery.

**Figure 2:**
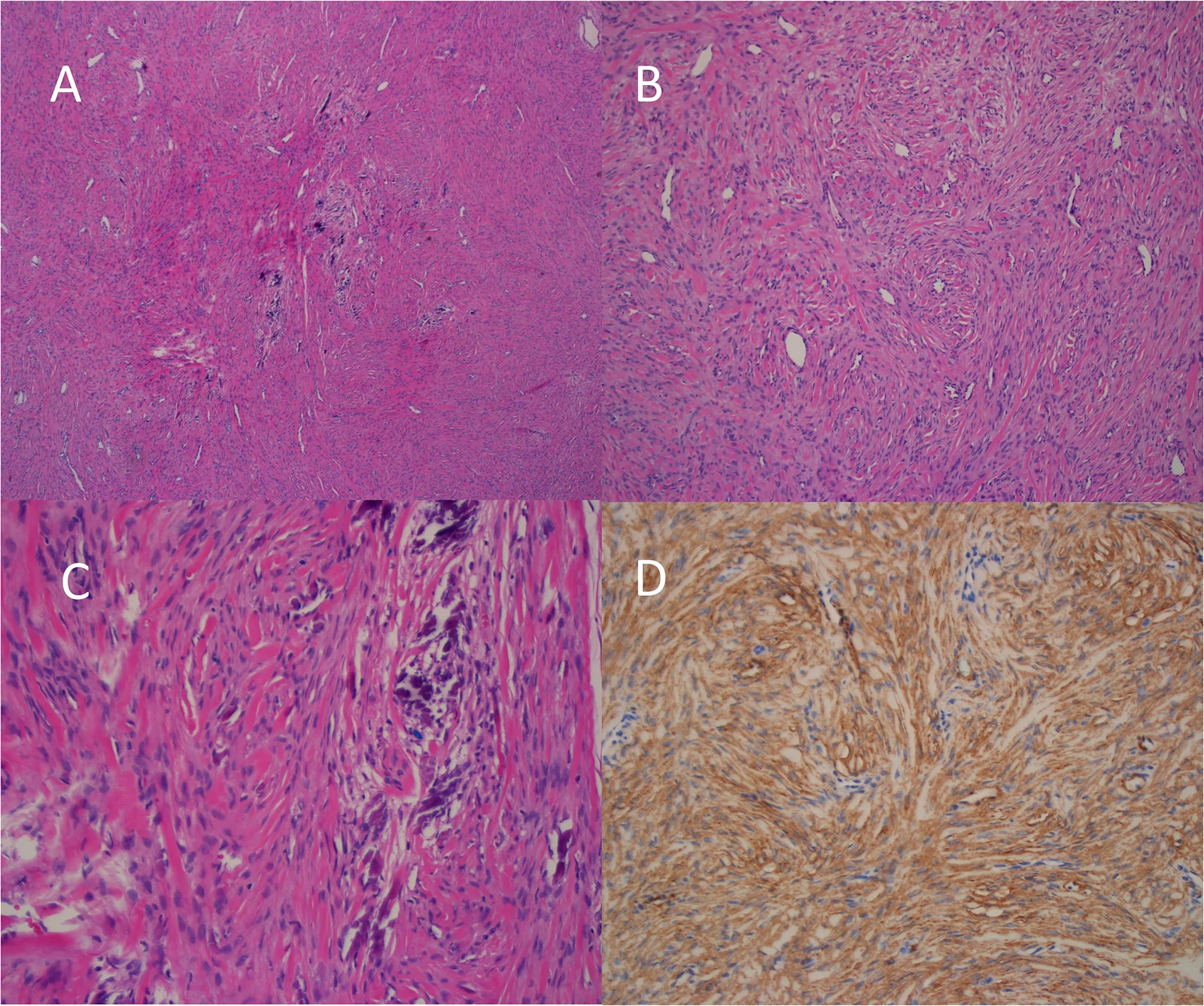
Histopathology demonstrating a well-circumscribed spindle cell lesion in collagenous stroma with dystrophic calcifications (Hematoxylin/Eosin at 40×, 100×, and 400× magnification shown in **(A–C)** respectively). Tumor cells are SMA-positive (D, 400× magnification).

The specimen was sent for frozen section analysis and permanent pathologic evaluation. The gross evaluation of the specimen revealed a well-circumscribed, firm white nodule. Upon sectioning, the cut surface showed a white, fibrous appearance. Frozen section of a representative portion demonstrated bland spindle cells without features of malignancy (**Figure 2**), and testis-sparing management was pursued following discussion with the family. The testis was returned to the hemiscrotum, the incision was closed, local anesthetic was administered, and the patient was discharged home in stable condition.

Frozen section and permanent histologic evaluation were concordant. Histologic examination demonstrated a well-circumscribed nodule within the paratesticular soft tissue composed of bland spindle cells arranged in a loose collagenous stroma, with associated dystrophic calcifications (**Figure 2**). Immunohistochemical analysis showed tumor cells positive for smooth muscle actin (SMA) and negative for desmin, myogenin, MyoD1, β-catenin, and S100. Anaplastic lymphoma kinase (ALK)-1 immunostaining was also negative, excluding IMT.

## Follow-up

At postoperative follow-up, the patient reported no interval complications and experienced no significant postoperative pain. He had returned to normal activities with normal oral intake, urination, and bowel movements. Scrotal ultrasound performed at 3 months was unremarkable, with no evidence of recurrence. The patient will continue surveillance with annual ultrasound for at least 5 years postoperatively.

## Discussion

Paratesticular tumors in prepubertal children are predominantly benign, underscoring the importance of accurate diagnosis to guide appropriate surgical management and avoid unnecessary orchiectomy (1, 2). Among benign paratesticular tumors, fibrous pseudotumor is the third most common entity after adenomatoid tumor and lipoma (2), but it is predominantly described in adults, with pediatric cases exceedingly rare (6). Myofibroblastic tumors, including myofibroma, are uncommon in the paratesticular region. Myofibroma is the most frequent fibrous tumor of childhood overall, yet testicular involvement is extremely rare, and isolated paratesticular lesions have not previously been reported (3). This rarity complicates differentiation from other spindle cell tumors, including fibrous pseudotumor and IMT.

Pediatric paratesticular spindle cell lesions can be challenging to distinguish clinically and radiographically. IMT, for example, is more often reported in adolescents and is typically characterized by inflammatory infiltrates and ALK positivity (6, 7). In the present case, histopathology supported a diagnosis of benign myofibroblastic lesion and excluded IMT.

Accurate intraoperative and histopathologic assessment allowed for testis-sparing excision, avoiding unnecessary orchiectomy. The patient tolerated the procedure well, with no postoperative complications, and will undergo surveillance with scrotal ultrasound every 3 months during the first year.

This case highlights the critical role of intraoperative frozen section in guiding testis-sparing management in indeterminate paratesticular masses (**Table 1**). To our knowledge, this represents one of the earliest presentations of a benign paratesticular myofibroblastic lesion in a toddler. Recognition of this rare entity and its distinguishing histologic and immunohistochemical features facilitate testis-sparing surgical management.

**Table 1:** Summary Table.

Stage of care	Summary of findings and actions
Presentation and initial evaluation	Asymptomatic 2-year-old boy with incidentally noted right scrotal mass. Ultrasound demonstrated a ~1.2-cm well-circumscribed extratesticular lesion near the epididymal head with internal vascularity; testes normal. Serum tumor markers (AFP, β-hCG, and LDH) were within normal limits.
Surveillance, preoperative assessment, and management decision	Serial ultrasounds over 3 months showed a stable ~1-cm lesion without concerning features. Persistent, mobile mass on exam. Because of the inability to definitively exclude malignancy, multidisciplinary consensus to proceed with inguinal exploration and intraoperative frozen section.
Operative findings and pathology	Inguinal exploration revealed a firm paratesticular mass arising from the tunica albuginea, excised with testis preservation. Frozen section showed bland spindle cells without malignant features. Final pathology confirmed a benign myofibroblastic lesion (SMA+, ALK−, and desmin−) with collagenous stroma and calcifications.
Postoperative course and follow-up	Uncomplicated recovery. At 3-month follow-up, ultrasound demonstrated no evidence of recurrence. Will continue with annual ultrasound surveillance for at least five postoperative years.

This report is limited by its single-patient design, which limits the generalizability of its findings to other pediatric patients with similar lesions. Although frozen section analysis and immunohistochemistry supported a benign diagnosis, the lack of broader molecular profiling may limit definitive characterization of this spindle cell lesion. In addition, follow-up duration is relatively short, and longer surveillance is needed to assess for recurrence and confirmation of outcomes.

## Conclusion

Paratesticular benign myofibroblastic lesions are extremely rare benign tumors in prepubertal children and should be considered in the differential diagnosis of paratesticular spindle cell lesions. Accurate histopathologic evaluation, supported by immunohistochemistry, is essential to rule out IMT, enabling testis-sparing surgery and avoiding unnecessary orchiectomy.

## Data Availability

The original contributions presented in the study are included in the article/supplementary material. Further inquiries can be directed to the corresponding author.
